# The Shot CH1 domain recognises a distinct form of F-actin during *Drosophila* oocyte determination

**DOI:** 10.1242/dev.202370

**Published:** 2024-04-02

**Authors:** Dmitry Nashchekin, Iolo Squires, Andreas Prokop, Daniel St Johnston

**Affiliations:** ^1^ The Gurdon Institute and the Department of Genetics, University of Cambridge, Tennis Court Road, Cambridge CB2 1QN, UK; ^2^ The University of Manchester, Manchester Academic Health Science Centre, Faculty of Biology, Medicine and Health, School of Biology, Manchester M13 9PT, UK

**Keywords:** Calponin homology domain, Cytoskeleton, Cell polarity, Gametogenesis, Germ cell, Spectraplakin

## Abstract

In *Drosophila*, only one cell in a multicellular female germline cyst is specified as an oocyte and a similar process occurs in mammals. The symmetry-breaking cue for oocyte selection is provided by the fusome, a tubular structure connecting all cells in the cyst. The *Drosophila* spectraplakin Shot localises to the fusome and translates its asymmetry into a polarised microtubule network that is essential for oocyte specification, but how Shot recognises the fusome is unclear. Here, we demonstrate that the actin-binding domain (ABD) of Shot is necessary and sufficient to localise Shot to the fusome and mediates Shot function in oocyte specification together with the microtubule-binding domains. The calponin homology domain 1 of the Shot ABD recognises fusomal F-actin and requires calponin homology domain 2 to distinguish it from other forms of F-actin in the cyst. By contrast, the ABDs of utrophin, Fimbrin, Filamin, Lifeact and F-tractin do not recognise fusomal F-actin. We therefore propose that Shot propagates fusome asymmetry by recognising a specific conformational state of F-actin on the fusome.

## Teaser

**Summary:** Actin filaments in a specific conformational state are formed during oocyte fate establishment in *Drosophila*, and they are recognised only by a subset of actin-binding proteins.

## INTRODUCTION

Both male and female gametes differentiate inside cysts of interconnected germ cells. Whereas all male cells in the cyst become sperm, only one or few of the female germ cells are specified to become oocytes in most animals (Lu et al., 2017; Pepling and Spradling, 1998; Lei and Spradling, 2013). Given that cells in the female germline cyst share cytoplasm through intercellular bridges, there must be specific mechanisms to select the future oocyte. In *Drosophila*, a polarised microtubule (MT) network extends throughout the cyst and directs the dynein-dependent transport of oocyte fate determinants into one cell (Li et al., 1994; Theurkauf et al., 1993). A similar mechanism could also be involved in oocyte specification in the mouse (Lei and Spradling, 2016; Niu and Spradling, 2022).

**Fig. 1.**
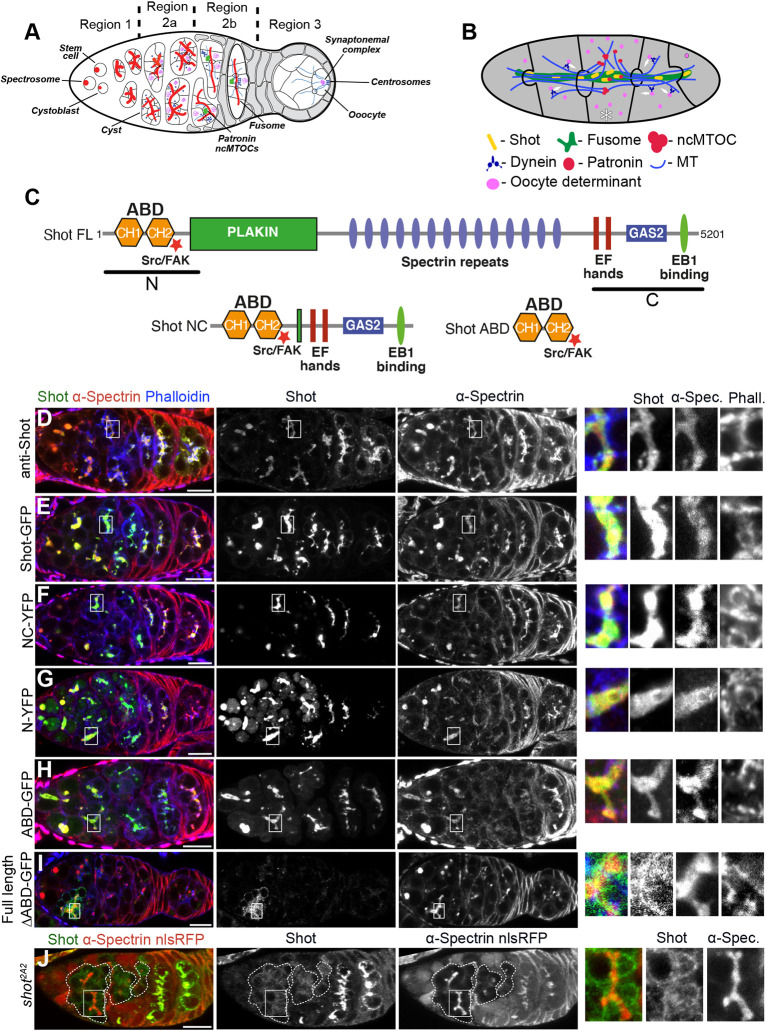
**Shot is recruited to the fusome by its ABD.** (A) Schematic of the *Drosophila* germarium showing germline cyst formation and oocyte specification. See text for further details. (B) Diagram of a germline cyst showing how Shot and Patronin translate fusome asymmetry into the polarised MT network that directs dynein transport of oocyte fate determinants into the prospective oocyte (asterisk). Schematics in A and B adapted from Nashchekin et al. (2021). (C) The domain structure of full-length (FL) Shot and Shot truncations. CH, calponin homology domain. (D-I) Localisation of endogenous Shot (D) and ectopically expressed full-length Shot-GFP (E), Shot-NC-YFP (F), Shot-N-YFP (G), Shot ABD-GFP (H) and Shot^ΔABD^ (I) in wild-type germaria. (J) Shot localisation in *shot^2A2^* mutant cysts. Mutant cysts are marked by dashed lines and are labelled by the absence of nuclear RFP (nlsRFP, red). An enlargement of a fusome (boxed area) is shown on the right. α-Spectrin marks the fusome. Phalloidin marks ring canals and the cell cortex. Scale bars: 10 µm.

In *Drosophila*, cyst formation and oocyte specification occur in the germarium at the anterior of the fly ovary (Fig. 1A). Oocyte determination starts when a germline stem cell divides asymmetrically to produce a cyst progenitor, a cystoblast, which goes through four rounds of incomplete division to produce a cyst of 16 cells connected by intercellular bridges called ring canals (de Cuevas et al., 1997). The cystoblast contains a spherical structure inherited from the stem cell called the spectrosome, which contains endoplasmic reticulum, spectrins and actin-binding proteins (Lighthouse et al., 2008). At each subsequent division, new spectrosomal material forms in the ring canal connecting the two daughter cells and this fuses with the pre-existing spectrosome to form the fusome, which becomes a branched structure extending into all 16 cells of the cyst (De Cuevas and Spradling, 1998; Lin et al., 1994). Because one cell inherits the original spectrosome/fusome from the cystoblast, this cell contains more fusomal material than the others and this ultimately specifies it as the pro-oocyte (De Cuevas and Spradling, 1998; Lin and Spradling, 1995).

The first step in the translation of fusome asymmetry into oocyte specification is the recruitment of the *Drosophila* spectraplakin Shot (Nashchekin et al., 2021; Roper and Brown, 2004). Shot in turn recruits the MT minus end-binding protein Patronin (CAMSAP in mammals) to the fusome, where Patronin stabilises microtubule minus ends (Goodwin and Vale, 2010; Jiang et al., 2014; Nashchekin et al., 2021). The slight excess of Patronin in the future oocyte is then amplified by the dynein-dependent transport of Patronin and microtubule minus ends along the stabilised microtubules into this cell, leading to the formation of non-centrosomal microtubule-organising centres (ncMTOCs) in the future oocyte. Finally, these ncMTOCs nucleate a polarised MT network that directs the transport of oocyte determinants into this cell (Bolívar et al., 2001; Grieder et al., 2000; Nashchekin et al., 2021) (Fig. 1B).

The spectraplakin Shot belongs to a conserved family of actin-microtubule crosslinkers that includes human dystonin and ACF7 (MACF1), which play important roles in cytoskeletal organisation during neurogenesis and in epithelia (Dogterom and Koenderink, 2019; Hahn et al., 2016; Lee and Kolodziej, 2002). Spectraplakins are characterised by an N-terminal actin-binding domain (ABD), a central long rod domain consisting of plakin and spectrin repeats and a C-terminal MT-binding module (Fig. 1C). The ABD of spectraplakins consists of tandem calponin homology domains, CH1 and CH2, and the MT-binding module is composed of the MT lattice-binding GAS2 domain and an unstructured C-terminal domain containing two SxIP motifs that interact with the MT plus end-binding protein EB1 (Applewhite et al., 2010; Honnappa et al., 2009; Sun et al., 2001). Although Shot transmits fusome asymmetry to Patronin localisation and the formation of the polarised MT network that specifies the oocyte, how Shot recognises the fusome is not known. Previous work suggested that the Shot ABD is not involved (Roper and Brown, 2004). Here, we show, however, that both the actin- and MT-binding domains are required for oocyte specification, consistent with the role of Shot in organising the polarised microtubule network. The Shot ABD is necessary and sufficient for localisation to the fusome and recognises a form of F-actin that differs from other F-actin networks in the cyst.

## RESULTS AND DISCUSSION

### The Shot ABD localises to the fusome

**Fig. 2.**
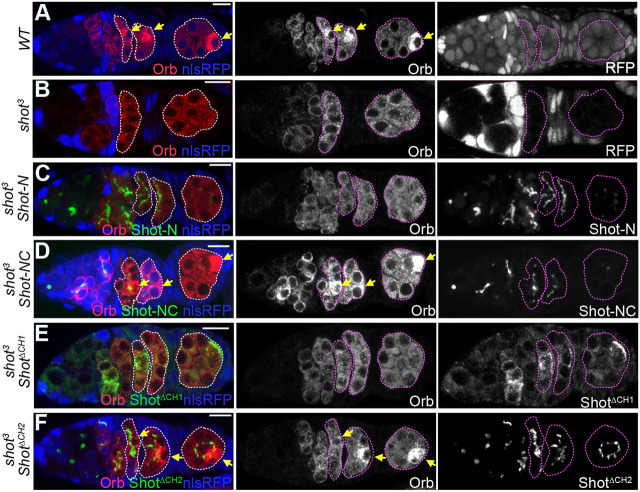
**The Shot CH1 and MT-binding domains are required for oocyte determination.** (A,B) The distribution of Orb in wild-type (WT; A) and *shot^3^* (B) germline clone mutant cysts marked by the loss of nlsRFP (blue). (C-F) The requirement of Shot domains for the oocyte determination. Germaria expressing Shot-N-YFP (C), Shot NC-YFP (D), Shot^ΔCH1^-GFP (E) and Shot^ΔCH2^-GFP (F) in *shot^3^* germline clones and stained for Orb (red). Arrows point to the future oocyte; cysts are marked by dashed lines; mutant cysts are labelled by the absence of nuclear RFP (nlsRFP, blue). Scale bars: 10 µm.

To determine which Shot domain(s) direct its localisation to the fusome, we expressed a mini-version of Shot lacking the central rod domain, Shot-NC (Fig. 1C). It has been previously shown that the NC version of ACF7 (a mammalian Shot homologue) partially substitutes for ACF7 function in cells (Wu et al., 2008). Like endogenous Shot (Fig. 1D) and the full-length Shot transgene (Fig. 1E), Shot-NC localised to the fusome, which is marked by α-spectrin (Fig. 1F, Fig. S1A). Thus, the fusome-binding activity of Shot resides in either its N- or C-terminal domains and the rod domain is dispensable for fusome localisation. Expression of the N- or C-terminal domains alone showed that Shot-N binds to the fusome in wild-type (Fig. 1G) and *shot* mutant (Fig. 2C) cysts, whereas Shot-C forms cytoplasmic foci and accumulates in one cell of the cyst in wild type (Fig. S1A,B), but not in *shot* mutant cysts (Fig. S1D), a pattern previously described for EB1 (Nashchekin et al., 2021). Live imaging of Shot-C-YFP in the germarium revealed that it forms EB1-like comets (Movie 1), suggesting that the C-terminal domain of Shot associates with MT plus ends, and that the MT lattice-binding GAS2 domain is in an inhibited conformation, as previously shown for the mammalian Shot homologue dystonin in Cos-7 cells (Kapur et al., 2012). Nevertheless, we decided to test whether the GAS2 domain has the potential to bind the fusome by expressing a portion of the Shot C-terminal domain containing the EF hand and the GAS2 domain that has strong MT binding activity (Maybeck and Roper, 2009). EF-GAS2-GFP localised to the fusome in the presence of endogenous Shot (Fig. S1A,B), but failed to do so in *shot* mutant cysts (Fig. S1C,D). Fusome-associated MTs are largely lost in the absence of Shot, indicating that EF-GAS2-GFP localises to the fusome by binding to Shot-dependent MTs (Roper and Brown, 2004). The GAS2 domain therefore cannot be responsible for the initial recruitment of Shot to the fusome.

From our results so far, we speculated that Shot recruitment to the fusome requires the Shot N-terminal region containing the two calponin homology domains that constitute the ABD. We therefore expressed a construct containing just the ABD of Shot fused to GFP and confirmed its localisation to the fusome (Fig. 1H, Fig. S1A). In contrast, full-length Shot lacking the ABD (Shot^ΔABD^) showed only a residual fusome localisation (Fig. 1I, Fig. S1A), which disappeared in the absence of endogenous Shot (Fig. S1D), similar to Shot C and Shot EF-GAS. Thus, the ABD of Shot is both necessary and sufficient for fusome localisation, presumably by interacting with fusome-associated F-actin. To confirm that Shot ABD is recruited to the fusome through the interaction with F-actin we took advantage of the *shot^2A2^* hypomorphic allele, which contains a point mutation in the actin-binding surface of the ABD (Chang et al., 2011; Nashchekin et al., 2016) and reduces Shot's interaction with the actin-rich cell cortex in epithelial cells and the oocyte (Nashchekin et al., 2016). Indeed, Shot showed a reduced localisation to the fusome in *shot^2A2^* mutant cysts (Fig. 1J).

### The actin- and MT-binding domains of Shot are required for the oocyte specification

The oocyte is not specified in the absence of Shot, leading to the formation of a follicle containing 16 nurse cells, but which domains of Shot are required for oocyte specification is not known (Roper and Brown, 2004). The cytoplasmic polyadenylation element binding factor Oo18 RNA-binding protein (Orb) is an early marker for oocyte specification that becomes concentrated in the oocyte in regions 2b and 3 in wild-type germaria, but is uniformly distributed in *shot* null mutant cysts (Lantz et al., 1994; Roper and Brown, 2004) (Fig. 2A,B). To determine whether the presence of the Shot actin- or MT-binding domains are sufficient for oocyte specification, we expressed Shot-N, Shot-C, Shot-NC and Shot-EF-GAS2 in *shot* null mutant cysts (Fig. 2C,D, Fig. S1D). Although Shot-N bound to the fusome in the absence of endogenous Shot, it did not rescue Orb localisation and oocyte determination (Fig. 2C). Neither Shot-C nor Shot-EF-GAS2 localised to the fusome or rescued oocyte specification in *shot* mutants (Fig. S1C,D). However, Shot-NC expression restored oocyte specification in the absence of full-length Shot (Fig. 2D). Thus, both the ABD and MT-binding modules are necessary for Shot function during oocyte determination, suggesting that Shot acts as an actin–microtubule cross-linker in this context.

It has been previously reported that the Shot ABD is not required for oocyte specification, because the oocyte is specified normally in cysts mutant for *shot^kakp1^*, a P-element insertion that is predicted to prevent the expression of CH1 domain-containing Shot isoforms (Roper and Brown, 2004). As shown above, however, the Shot ABD is essential for Shot localisation to the fusome and oocyte determination. To resolve this contradiction, we tested the requirement for the Shot CH1 and CH2 domains in oocyte specification by expressing Shot^ΔABD^, Shot^ΔABD^-Lifeact, Shot^ΔCH1^ and Shot^ΔCH2^ in *shot* mutant cysts (Fig. 2E,F, Fig. S1D). Shot truncations lacking the CH1 domain did not rescue oocyte specification, nor did substituting the Shot ABD with the actin-binding activity of Lifeact (Fig. 2E, Fig. S1D). Only *shot* mutant cysts expressing Shot^ΔCH2^ maintained Orb localisation and specified the oocyte (Fig. 2F). Thus, the CH1 domain of the Shot ABD is essential for oocyte specification. Because the *shot^kakp1^* mutant does not affect oocyte determination, we assume that this P-element insertion does not disrupt the expression of CH1-containing Shot isoforms in the germ line, although it does so in somatic tissues (Roper and Brown, 2004).

Previously, it has been proposed that Shot binds the fusome with an unidentified domain and uses its GAS2 domain to bind and stabilise MTs (Roper and Brown, 2004). Based on our results, we propose an alternative model for Shot function in oocyte determination whereby it works as a classical actin–MT cytolinker by recognising fusomal F-actin with its ABD and using its C-terminal domain to attach MTs to the fusome. Which part of the MT module is involved in this process is an open question. Shot-EF-GAS2 can recognise fusomal MTs, but expression of the whole Shot C-terminal domain showed that the GAS2 domain is not exposed and Shot C interacts only with MT plus ends through its EB1-binding motifs. Thus, Shot may guide the growth of MT plus ends along the fusome in a similar manner to that described for ACF7 in migrating cells (Kodama et al., 2003; Wu et al., 2008). The role of Shot in stabilising MTs on the fusome could also be indirect, as we recently showed that Shot is required for the fusome localisation of the MT minus-end stabilising protein Patronin/CAMSAP (Nashchekin et al., 2021). Moreover, fusome-associated MTs are unstable in *patronin* mutant cysts even though Shot is still present. How the Shot C terminus recruits microtubules to the fusome will therefore require further study.

### Shot recognises a distinctive form of F-actin on the fusome

**Fig. 3.**
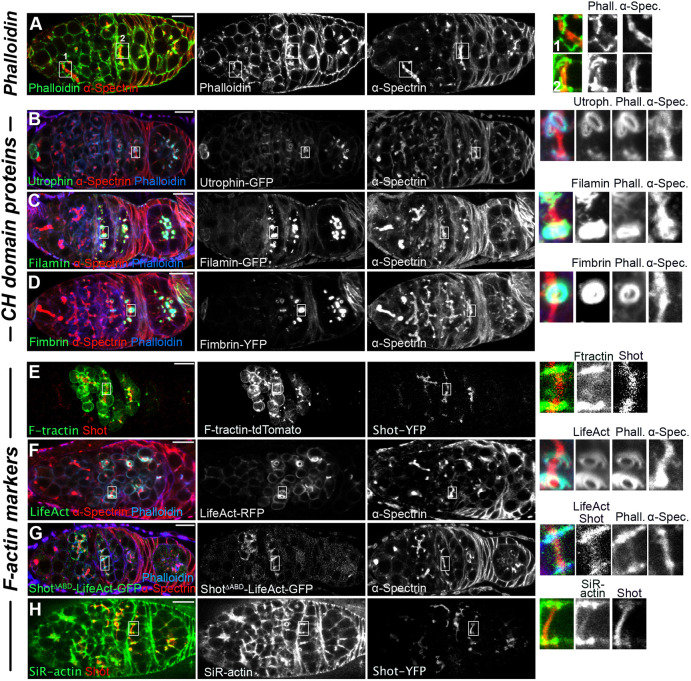
**Recognition of fusomal F-actin by commonly used F-actin labelling reagents and CH domain proteins.** (A) Detection of F-actin on the fusome**.** Confocal image of a germarium stained for 21 h with FITC Phalloidin (green) and anti-α-Spectrin antibody (red). (B-D) Localisation of the CH domain proteins (green) utrophin ABD-GFP (B), Filamin-GFP (C) and Fimbrin-YFP (D) in cysts stained for α-Spectrin (red) to label the fusome and cell cortices and phalloidin (blue), which labels the ring canals. Utrophin ABD-GFP was exogenously expressed, whereas Filamin and Fimbrin are endogenously expressed protein-trap lines. (E) Confocal image of a living germarium expressing F-tractin-tdTomato (green) and Shot-YFP (red). (F,G) Confocal images of germaria expressing Lifeact-RFP (green) (F) and Shot^ΔABD^-Lifeact-GFP (G) stained for α-Spectrin (red) and phalloidin (blue). (H) Confocal image of a living germarium expressing Shot-YFP (red) and incubated for 30 min with SiR-actin (green). The right-hand panels show enlargements of the boxed regions of the fusome in the left-hand panels. Scale bars: 10 µm.

Several actin-binding proteins have been identified as components of the fusome, including β-Spectrin, Hts (also known as Adducin), Tropomodulin and Shot (De Cuevas et al., 1996; Lighthouse et al., 2008; Lin et al., 1994; Roper and Brown, 2004). However, actin has never been detected in the fusome, and under standard conditions phalloidin does not label the fusome, staining only the ring canals and the cell cortex (Warn et al., 1985) (Fig. 1D-I). In contrast, Actin-GFP weakly localises to the fusome, raising the possibility that the absence of phalloidin staining might be misleading (Fig. S2A). We therefore tested for the presence of endogenous actin in the fusome by performing a prolonged, 21 h staining with phalloidin, which produced a weak but consistent fusomal signal (Fig. 3A). We conclude that the fusome does contain F-actin, but not in a form that can be easily detected by phalloidin. Given that the phalloidin–actin interaction is sensitive to the structure of F-actin filaments (McGough et al., 1997), fusomal F-actin may exist in a distinct conformation that can be bound by the Shot ABD but only weakly by phalloidin.

Both β-Spectrin and Shot are members of a large family of actin-binding proteins with ABDs formed by tandem CH domains (Korenbaum and Rivero, 2002; Yin et al., 2020) (Fig. S2B). To test whether other CH domain proteins recognise fusomal F-actin, we analysed the distribution of overexpressed human utrophin ABD and endogenously tagged Filamin and Fimbrin. None of these CH domain proteins recognised fusomal F-actin and they mainly concentrated at the ring canals in fixed (Fig. 3B-D) and live (Fig. S2C) samples. F-tractin, Lifeact and SiR-actin are other commonly used F-actin markers (Melak et al., 2017), recognising a variety of F-actin structures (Belin et al., 2014; Riedl et al., 2008; Schell et al., 2001; Spracklen et al., 2014). Neither F-tractin nor Lifeact localised to the fusome, instead mainly localising to the cell cortex and ring canals (Fig. 3E,F, Fig. S2C). Moreover, substituting the Shot ABD with the Lifeact sequence (Shot^ΔABD^-Lifeact) did not restore fusome recognition to full-length Shot (Fig. 3G, Fig. S1A). By contrast, SiR-actin weakly localised to the fusome (Fig. 3H). Thus, fusomal F-actin has a distinctive conformation that is only recognised by a subset of actin-binding proteins/reagents. The Shot ABD must therefore have structural features that allow it to bind preferentially to fusomal F-actin and to distinguish it from other F-actin structures in the cyst. This does not preclude Shot ABD binding to other forms of F-actin, because Shot re-localises to the cell cortex and ring canals in *hts* and *α-spectrin* mutant cysts that lack the fusome (De Cuevas et al., 1996; Lin and Spradling, 1995) (Fig. S3). Shot also binds to actin filaments in the ring canal baskets that form at later stages of oogenesis when the fusome is disassembled (Lu et al., 2021).

**Fig. 4.**
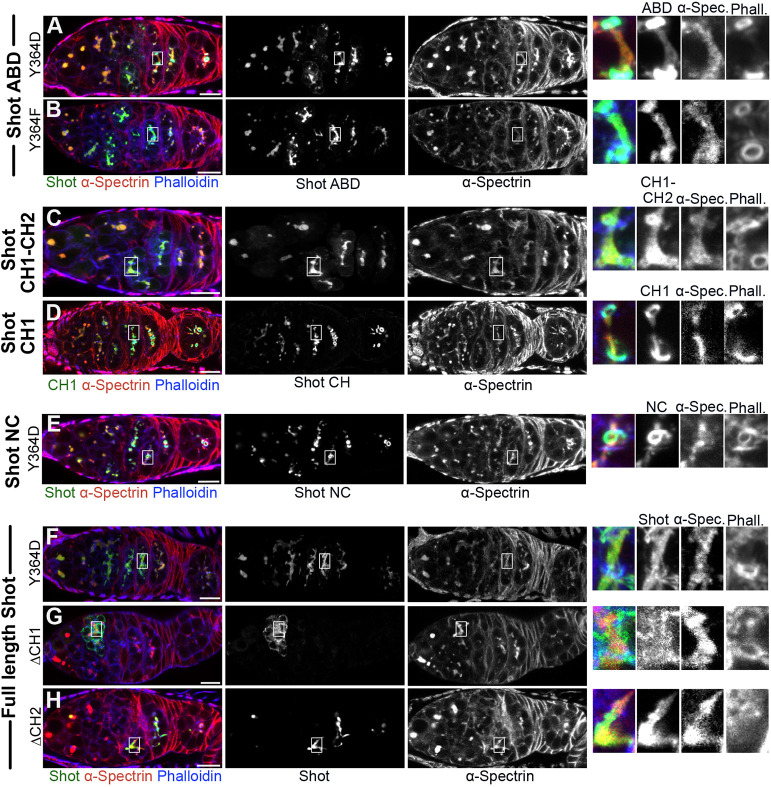
**The Shot CH1 domain recognises the fusome.** (A,B) Germaria expressing GFP-ABDY364D (A) and GFP-ABDY364F (B), illustrating the effects of the phosphomimetic Y364D and non-phosphorylatable Y364F mutations on the localisation of the Shot ABD. (C,D) Germaria expressing GFP-CH1-CH2 (C) and GFP-CH1 (D), illustrating the role of CH2 in regulating the actin-binding properties of CH1. (E,F) Germaria expressing Shot-NCY364D-YFP (E) and ShotY364D-GFP (F), illustrating the effects of the phosphomimetic Y364D mutation on the localisation of Shot NC and full-length Shot. (G,H) Germaria expressing Shot^ΔCH1^-GFP (G) and Shot^ΔCH2^-GFP (H), illustrating the role of CH domains in Shot localisation on the fusome. The right-hand panels show enlargements of the boxed regions of the fusome in the left-hand panels. α-Spectrin (red in the left-hand panels) marks the fusome. Phalloidin (blue) marks the ring canals and cell cortex. Scale bars: 10 µm.

The spatial arrangement of tandem CH1 and CH2 domains can regulate their actin-binding activity, as the CH2 domain can sterically block some of the actin-binding surfaces on the CH1 domain (Bañuelos et al., 1998; Galkin et al., 2010; Iwamoto et al., 2018). It has been proposed that phosphorylation of a conserved Tyr located at the end of the CH2 domain facilitates the formation of an ‘open’ conformation of the ACF7 ABD and enhances F-actin binding (Yue et al., 2016) (Fig. 1C). To test whether Shot binding to the fusome is regulated by Tyr phosphorylation, we expressed Shot ABDs containing phosphomimetic and non-phosphorylatable versions of Tyr364 (ABD Y364D and ABD Y364F, respectively). Whereas ABD Y364F only bound to fusomal F-actin (Fig. 4B), ABD Y364D bound to the fusome and ring canals in region 2a of the germarium and mostly relocalised to ring canals in region 2b (Fig. 4A). This suggests that shifting the equilibrium towards an open state of the CH domains alters the specificity of the Shot ABD for different forms of F-actin, rather than working as a simple on/off switch. Because isolated ABDs might behave differently from the full-length protein, we introduced the Tyr364 mutations into Shot-NC and full-length Shot. Whereas the Shot-NC Tyr364 mutants behaved like the corresponding ABD mutants, the localisation of the full-length Shot was not affected by the Y364D mutation (Fig. 4E,F, Fig. S2D). Thus, sequences in the rod domain somehow restore the specificity of the Y364D ABD for fusomal actin.

To examine the role of CH2 in regulating the actin-binding properties of CH1, we compared the localisation of CH1 alone with that of the full ABD containing CH1 and CH2. Whereas CH1–CH2 exclusively localised to the fusome (Figs 1H and 4C), CH1 alone recognised F-actin on the fusome and ring canals (Fig. 4D). Thus, removing the CH2 domain has the same effect as the Y364D mutation in making the binding of CH1 to F-actin more promiscuous. According to recent structural studies, CH1–CH2 binds to F-actin filaments in an open conformation, with CH1 sitting in the groove between subdomains 1 and 2 of an actin monomer and CH2 oriented away from the filament (Galkin et al., 2010; Iwamoto et al., 2018; Kumari et al., 2020). This implies that binding of CH1 to F-actin breaks multiple interactions between the CH1 and the CH2 (Galkin et al., 2010; Harris et al., 2019, 2020; Iwamoto et al., 2018; Kumari et al., 2020; Yue et al., 2016). We can envisage two models for how CH2 controls the specificity of CH1's actin binding. CH2 in the closed conformation of the ABD may itself bind weakly to the specific form of actin on the fusome and thereby increase the avidity of the initial interaction of CH1–CH2 with fusomal actin. This increased avidity would then ensure that the Shot ABD binds preferentially to fusomal F-actin, which therefore outcompetes other F-actin in the cyst for Shot binding*.* Alternatively, CH2 may mask some actin-binding surfaces of CH1 in the ‘closed’ conformation of the ABD, allowing the latter to bind fusomal actin with higher affinity than other forms of actin. In another words, the presence of CH2 may increase the affinity of the ABD for fusomal actin or decrease its affinity for other forms of actin.

We also tested the role of each CH domain in the context of the full-length protein by analysing the localisation of full-length Shot lacking CH1 (Shot^ΔCH1^) or CH2 (Shot^ΔCH2^). Shot^ΔCH2^ localised to the fusome normally (Fig. 4H, Fig. S1A), whereas deletion of CH1 abolished fusome binding, leading to cytoplasmic localisation (Fig. 4G, Fig. S1A). The same pattern was observed in the absence of endogenous Shot (Fig. 2E,F). These results indicate that the CH1 domain mediates Shot binding to the fusome and that it can recognise a specific F-actin conformation that is invisible to several other CH-domain proteins and actin-binding molecules. This result also implies that specific binding to fusomal F-actin by Shot, which depends on CH2 in the isolated ABD, does not require CH2 in the context of the full-length protein.

Structural studies on the interaction of CH domains with F-actin have revealed that CH1 interacts with two adjacent actin monomers and is sensitive to the torque/helicity of F-actin filaments (Hanein et al., 1998; Harris et al., 2020; Iwamoto et al., 2018; Kumari et al., 2020). Changes in the helical twist of F-actin filaments can be caused by mechanical tension, by interactions with actin-binding proteins (Harris et al., 2020, 2018; Jégou and Romet-Lemonne, 2020), or by the bending of F-actin filaments promoted by differences in the nucleotide states of actin monomers (Oosterheert et al., 2022; Reynolds et al., 2022). It is therefore possible that CH1 domains of different actin-binding proteins are predisposed to bind F-actin filaments with specific helical twists, which could explain their distinct, but partly overlapping localisation patterns (Harris et al., 2020; Jégou and Romet-Lemonne, 2021; Washington and Knecht, 2008).

Although the Y367D mutation and the removal of CH2 lead to promiscuous binding of the Shot ABD to both the fusome and other F-actin in the cyst, they have no effect on the specific localisation of full-length Shot to the fusome. This indicates that some region of the full-length protein can substitute for CH2 in the ‘closed’ conformation of the ABD. This is most likely part of the rod domain given that the Y367D mutation still causes promiscuous actin binding in the context of Shot-NC (Fig. 4E). As proposed for CH2 in the context of the ABD alone, this rescue by the rod domain could be due to an interaction between the rod and CH1 that masks some actin-binding surfaces to reduce its affinity for nonfusomal actin. Because the rod is very long, it is also possible that this effect is indirect and acts by allowing the intramolecular interaction between the C-terminal EF hands and CH1 to achieve the same effect (Applewhite et al., 2013). Alternatively, the rod domain could increase the avidity of the interaction of Shot with the fusome by binding to some other fusomal component. Indeed, Shot has been proposed to bind to α-Spectrin, which is enriched on the fusome (De Cuevas and Spradling, 1998; Khanal et al., 2016; Lin et al., 1994). Because full-length Shot^ΔABD^ does not localise to the fusome, the rod domain is unlikely to function as a strong fusome-binding domain itself, but may contribute to avidity in a similar way to that proposed above for CH2.

Our evidence suggests that fusome asymmetry is propagated to organisation of MTs in the *Drosophila* female germline cyst by the formation of a distinct type of F-actin on the fusome, which then recruits the spectraplakin Shot. How fusomal F-actin is formed and the structural basis for its recognition by actin-binding proteins remain to be determined. Considering that Shot is a conserved actin-binding protein, it is possible that a similar mechanism is used in other contexts where F-actin filaments in a specific mechanical state are recognised by only a subset of actin-binding proteins.

## MATERIALS AND METHODS

### *Drosophila* stocks

The following previously described *Drosophila melanogaster* mutant alleles and transgenic lines were used: FRTG13 *shot^3^* (Roper and Brown, 2004), *hts^1^* and *hts^01103^* (Yue and Spradling, 1992), *α-spectrin^e2-26^* (Hülsmeier et al., 2007), UAS-GFP-Actin42A (Roper et al., 2005), UAS-GFP-Shot EF-GAS2 (Maybeck and Roper, 2009); UAS-Shot^ΔABD^-GFP, UAS-Shot^ΔCH1^-GFP, UAS-Shot^ΔCH2^-GFP and UAS-Shot^ΔABD^-Lifeact-GFP (Qu et al., 2022), Shot-YFP (Nashchekin et al., 2016), UAS-F-tractin-tdTomato (Spracklen et al., 2014), Filamin-GFP trap line (gift from K. Röper, MRC-LMB, UK), Fimbrin-YFP (Cambridge Protein Trap Insertion line 100066; Lowe et al., 2014), sqh>Utrophin ABD-GFP (Rauzi et al., 2010). UAS-Shot-GFP, UAS-ShotY364F-GFP, UAS-ShotY364D-GFP, UAS-Shot-NC-YFP, UAS-Shot-NCY364D-YFP, UAS-Shot-NCY364F-YFP, UAS-Shot-N-YFP, UAS-Shot-C-YFP, UAS-GFP-Shot ABD, UAS-GFP-Shot ABDY364F, UAS-GFP-Shot ABDY364D, UAS-Lifeact-RFP were generated for this study.

### *Drosophila* genetics

Germline clones of *shot^3^ and α-spectrin^e2-26^* were induced by incubating larvae at 37°C for 2 h per day over a period of 3 days. Clones were generated with FRT G13 nlsRFP and FRT 2A nlsRFP (Bloomington *Drosophila* Stock Center) using the heat-shock Flp/FRT system (Chou and Perrimon, 1992). Germline expression of UAS transgenes was induced by nanos>Gal4. All transgenes were expressed in a wild-type background unless otherwise specified.

### Molecular biology

To generate pUASP Shot-GFP, three fragments of the *shot* RE cDNA were amplified from pUAST Shot-GFP (Lee and Kolodziej, 2002) and cloned together with EGFP into the pUASPattb vector. pUASPattb-Shot-NC-YFP was generated by PCR, amplifying fragments from pUAST Shot-GFP corresponding to the first 520 aa (Shot-N) and last 462 aa (Shot-C) of Shot PE, cloning them together into pUASP-YFP-Cterm and then re-cloning into pUASPattb*.* pUASPattb-Shot-N-YFP and pUASPattb-Shot-C-YFP were generated by amplifying Shot-N or Shot-C fragments from pUASPattb-Shot-NC-YFP and cloning them together with YFP into pUASPattb. Shot ABD cDNA (corresponding to 146-368 aa of Shot PE) was amplified from pUASPattb-Shot-NC-YFP and cloned together with EGFP into pUASP-attb to generate pUASP-attb GFP-Shot ABD. The Q5 Site-Directed Mutagenesis Kit (New England BioLabs) was used to generate pUASPattb-GFP-Shot ABDY364F, pUASPattb-GFP-Shot ABDY364D, pUASPattb-Shot-NCY364D-YFP and pUASPattb-Shot-NCY364F-YFP. Shot-N with the Y364F or Y364D mutations was amplified from pUASPattb-Shot-NCY364F or pUASPattb-Shot-NCY364D, respectively, and cloned together with two fragments covering the rest of Shot RE cDNA and EGFP into pUASP-attb to generate pUASPattb-ShotY364F-GFP and pUASPattb-ShotY364D-GFP. pUASP-Lifeact-tagRFP was generated according to Riedl et al. (2008). NEBuilder HiFi DNA Assembly (New England BioLabs) was used for most of the cloning. Primer sequences are listed in Table S2.

### Immunohistochemistry

Ovaries were fixed for 20 min at room temperature in 4% paraformaldehyde and 0.2% Tween 20 in PBS. Ovaries were then blocked with 1% bovine serum albumin (BSA) in PBS with 0.2% Tween 20 for 1 h at room temperature. Ovaries were incubated with the primary antibody for 16 h with 0.1% BSA in PBS with 0.2% Tween 20 at 4°C and for 4 h with the secondary antibody at room temperature. For detection of fusomal F-actin, ovaries were fixed as above and were then incubated with FITC Phalloidin (1:300, Sigma, P5282) for 21 h at room temperature in PBS with 0.2% Tween 20 and 0.1% BSA. We used the following primary antibodies: guinea pig anti-Shot at 1:1000 (Nashchekin et al., 2016), mouse anti-Orb at 1:10 (DSHB Hybridoma Products 4H8 and 6H4; deposited by P. Schedl), mouse anti-α-Spectrin at 1:200 (DSHB Hybridoma Product 3A9; deposited by D. Branton and R. Dubreuil), rabbit anti-β-Spectrin at 1:200 (Byers et al., 1989), SiR-actin (1:100, Spirochrome, SC001). Secondary antibodies conjugated with Alexa Fluor dyes (Thermo Fisher Scientific, A11031, 21236, A21450 and A31571) were used at 1:1000.

### Imaging

Fixed preparations were imaged using a Leica SP8 (63×/1.4 HC PL Apo CS Oil) confocal microscope. Germaria were imaged by collecting 10-15 *z*-sections spaced 0.5 µm apart*.* For live imaging, ovaries were dissected and imaged in Voltalef oil 10S (VWR International) on a Leica SP5 confocal microscope (63×/1.4 HCX PL Apo CS Oil) or on an Olympus IX81 inverted microscope with a Yokogawa CSU22 spinning disc confocal imaging system (100×/1.3 NA Oil UPlanSApo). Images were collected with Leica LAS AF software or MetaMorph and processed using ImageJ. The images are projections of several *z*-sections. JACoP plug-in for ImageJ (Bolte and Cordelières, 2006) was used to quantify co-localisation between fusome and various Shot transgenes.

### Reproducibility of experiments and statistical analyses

Images are representative examples from at least three independent repeats for each experiment*.* The number of *shot^3^* mutant cysts (region 2b to 3) with restored Orb localisation after the expression of a rescue transgene were as follows: Fig. 2A, wild-type cysts (50/50); Fig. 2B, *shot^3^* mutant cysts without a transgene (0/30); Fig. 2C, Shot-N (0/21); Fig. S1D, Shot-C (0/28); Fig. S1D, Shot EFGAS (0/18); Fig. 2D, Shot-NC (25/26); Fig. S1D, Shot^ΔABD^ (0/14); Fig. S1D, Shot-Lifeact (0/19); Fig. 2E, Shot^ΔCH1^ (0/16); and Fig. 2F, Shot^ΔCH2^ (21/21). The chi-square test was used to test whether values were significantly different between wild-type and *shot^3^* mutant cysts. The number of cysts analysed to determine the localisation of various Shot transgenes and actin-binding reagents is summarised in Table S1. *P*<0.01 was considered to be statistically significant. No statistical methods were used to predetermine sample size, the experiments were not randomised, and the investigators were aware of group allocation during experiments and outcome assessment.

## Supplementary Material



10.1242/develop.202370_sup1 Supplementary information
