# Supplementary material for: The Shot CH1 domain recognises a distinct form of F-actin during Drosophila oocyte determination
Source: Development. 2024 Apr 2;151(7):dev202370. doi: 10.1242/dev.202370 (PMC11058685; doi:10.1242/dev.202370)
Supplement: Supplementary information [file develop-151-202370-s1.pdf]

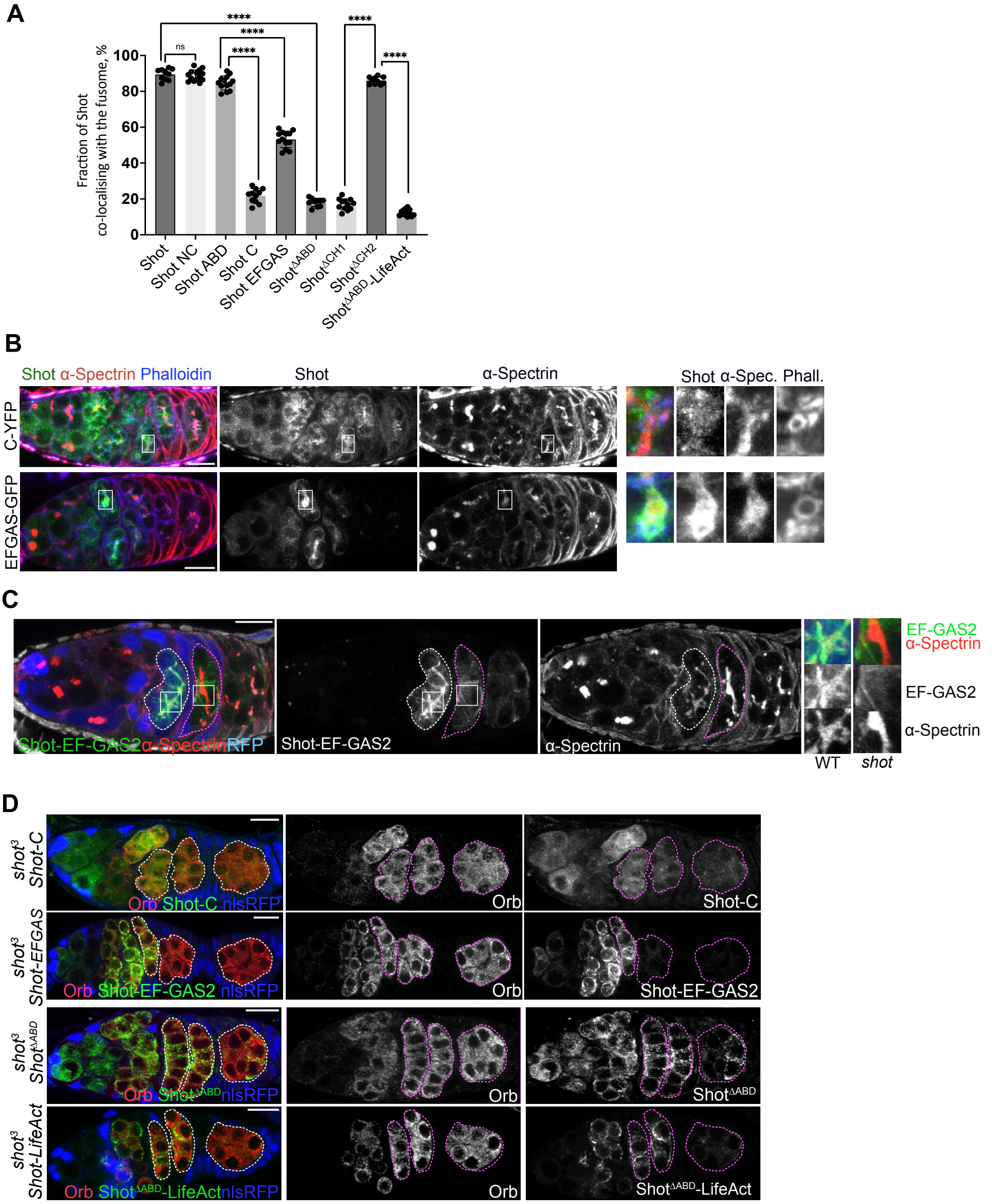

Fig. S1. (A) Quantification of fusome and Shot co-localisation in germaria expressing various Shot transgenes and stained with anti-α-Spectrin to label the fusome. Manders' co-localisation coefficient is measured using the JACoP plug-in for ImageJ (Bolte and Cordelières, 2006). Data are means with SD. Mann-Whitney test, **** $P < 0.0001$. (B) Ectopically expressed Shot-EF-GAS2-GFP localises to the fusome, whereas Shot C-YFP does not. An enlargement of a fusome is shown on

the right. α-Spectrin marks the fusome. Phalloidin marks ring canals and the cell cortex. (C) Shot-EF-GAS2-GFP does not localise to the fusome in the absence of endogenous Shot. A germarium expressing Shot-EF-GAS2-GFP (green) in wild type and *shot*^{3} mutant cysts. An enlarged view of a fusome is shown on the right. α-Spectrin (red in left panel) marks the fusome. (D) Shot-C-YFP, Shot-EF-GAS2-GFP, Shot$^{\Delta ABD}$-GFP and Shot$^{\Delta ABD}$-LifeAct-GFP do not rescue oocyte determination in *shot*^{3} germline clones. Cysts are marked by dashed lines; mutant cysts are labelled by the absence of nuclear RFP (nlsRFP; blue). Scale bars, 10μm.

Development • Supplementary information

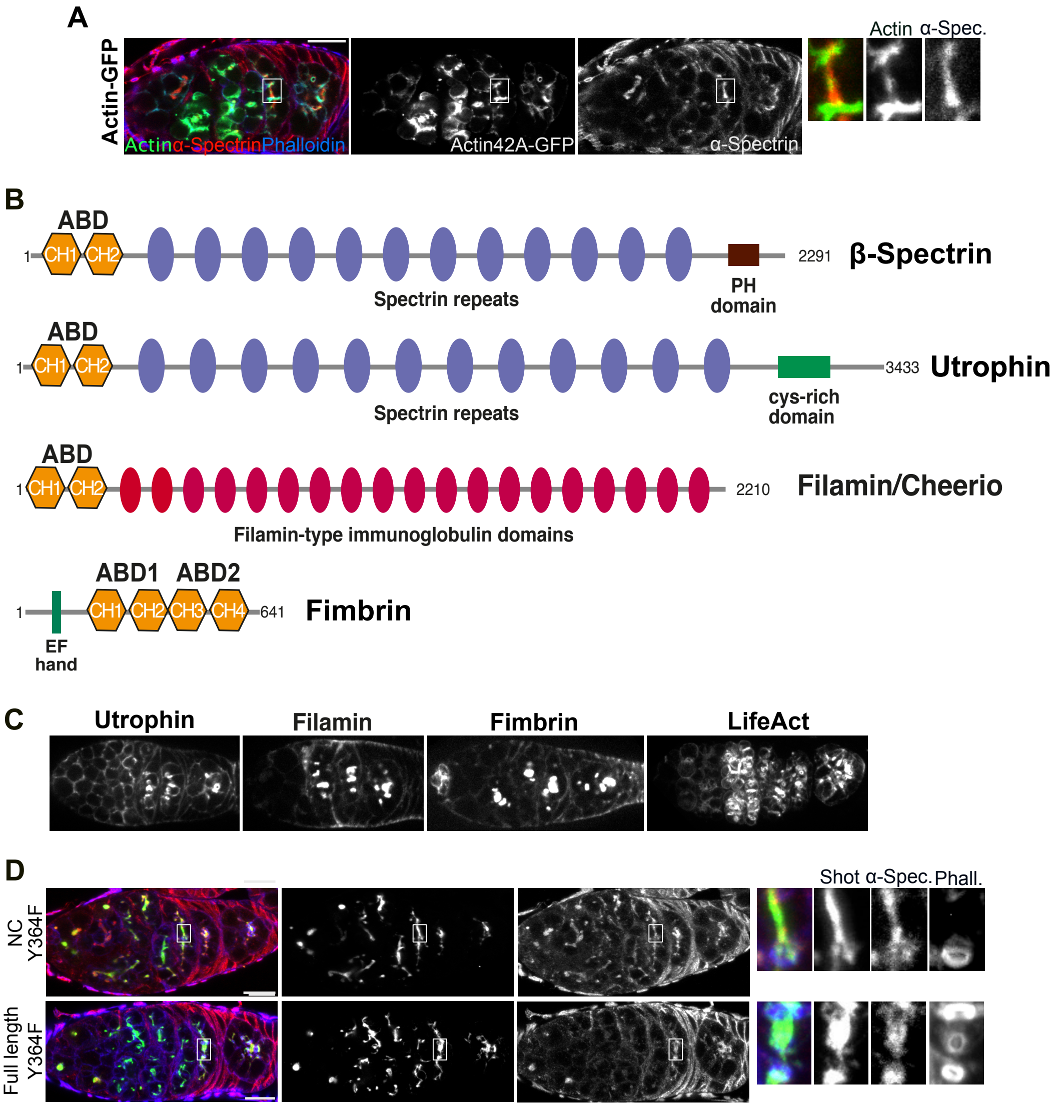

Fig. S2. (A) Actin-42A-GFP localises to the fusome. A germarium expressing Actin-42A-GFP (green) stained with anti-α-Spectrin antibody (red) to mark the fusome. The righthand panel shows an enlarged view of the fusome. (B) Diagrams showing the domain structure of actin-binding proteins with calponin homology (CH) domains. ABD, actin-binding domain. (C) Live germaria expressing Untrophin ABD-GFP, Filamin-GFP, Fimbrin-YFP and LifeAct-RFP. (D) The non-phosphorylatable mutation in Shot Tyr364 does not affect Shot NC and full length Shot localisation. Germaria expressing Shot-NC Y364F-YFP (top) and Shot Y364F-GFP (bottom). The righthand panels show enlargements of a fusome region. α-Spectrin (red in the lefthand panels) marks the fusome. Phalloidin (blue) marks the ring canals and cell cortex. Scale bars, 10µm.

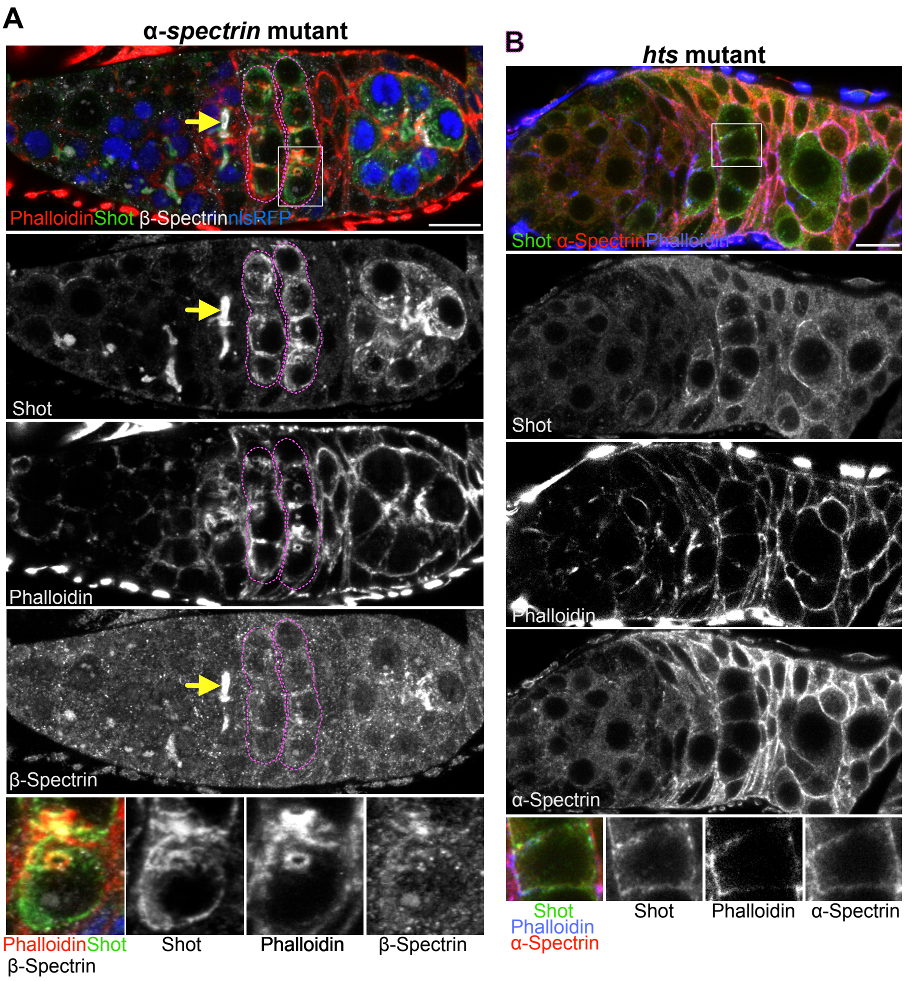

Fig. S3. Shot localises to the cell cortex and ring canals in *α-spectrin* and *hts* mutant cysts. (A) A germarium with *α-spectrin* germline clones stained with anti-Shot (red), Phalloidin (blue) and anti-β-Spectrin (white) to label the fusome. Mutant cysts are labelled by the absence of nuclear RFP (nlsRFP; blue). *α-spectrin* mutant cysts are marked by dashed lines. Arrows point to the fusome in a wild type cyst. (B) A germarium from a *hts* mutant female stained with anti-Shot (red), Phalloidin (blue) and anti-α-Spectrin (red) to label the fusome. Scale bars, 10μm.

Table S1. Localisation to the fusome and ring canals

Name of the protein	Localisation to the fusome / number of cysts analysed	Localisation to ring canals / number of cysts analysed	Number of germaria analysed	Expression in *shot*[3] mutant cysts restores oocyte specification
Shot FL	63/63	0/63	11	–
Shot NC	71/71	0/71	10	Yes
Shot N	66/66	0/66	10	No
Shot C	0/42	0/42	10	No
Shot EF-GAS2	32/32	0/32	8	No
Shot ABD	58/58	0/58	10	–
$Shot^{\Delta ABD}$	0/34	0/34	16	No
$Shot^{\Delta ABD}$-LifeAct	0/41	5 /41	12	No
$Shot^{\Delta CH1}$	0/31	0/31	10	No
$Shot^{\Delta CH2}$	47/47	0/47	10	Yes
Shot Y364D	74/74	0/40 (region 2a)<br>0/34 (region 2b)	10	_
Shot Y364F	45/45	0/25 (region 2a)<br>0/20 (region 2b)	10	_
Shot NC Y364D	49/49	10/27(region 2a)<br>22/22(region 2b)	10	_
Shot NC Y364F	45/45	0/24 (region 2a)<br>0/21(region2b)	10	_
Shot ABD Y364D	55/55	14/31(region 2a)<br>24/24(region2b)	10	_
Shot ABD Y364F	53/53	0/28 (region 2a)<br>0/25 (region 2b)	10	_
Shot CH1	53/53	13/29 (region 2a)<br>24/24 (region 2b)	10	_
Utrophin ABD	0/50	50/50	10	–
Filamin	0/51	51/51	10	–
Fimbrin	0/42	42/42	10	–
F-tractin	0/49	49/49	12	–
LifeAct	0/42	42/42	8	–
SiR-actin	41/41	41/41	8	–

Table S2. List of Primers

PRIMER NAME	SEQUENCE
Shot1F	CCCGGGGATCAGATCCGCATGACATCGCAT
Shot1R	CAATTCGGGCCTCAAGATCGGTAAGCTTCTGATCTACATG
Shot2F	CTTACCGATCTTGAGGCCCGAATT
Shot2R	CTGGGCGGCTACATCGGCAAG
Shot3F	GCCGATGTAGCCGCCCAGCTG
Shot3R	AACGTTCGAGGTCGACTCTAGAGTTACTTGTACAGCTCGTCCATGCCG
ShotNC1F	ACGTGGTACCATTATGACATCGCATTCCT
ShotNC1R	ACGTTCTAGAAAGATCGGTAAGCTTCTG
ShotNC2F	ACGTCTCGAGCTTCTGTATCTGAAG
ShotNC2R	ACGTTCTAGATAGCCGGAATGG
ShotN1F	CCCGGGGATCAGATCCGCATGACATCGCATTCCTACTATAAAGACCG
ShotN1R	GCCCTTGCTCACAAGATCGGTAAGCTTCTGATCTACATGCTTGATCT
ShotN2F	TTACCGATCTTGTGAGCAAGGGCGAGGAG
ShotN2R	AACGTTCGAGGTCGACTCTAGAGCTTGTACAGCTCGTCCATGCC
ShotC1F	CCCGGGGATCAGATCCGCATGCTTCTGTATCTGAAGGATGTGGAGCG
ShotABDF1	CCCGGGGATCAGATCCGCATGGTGAGCAAGGGCGAGGAG
ShotABDR1	GCATCGCGGGCGGCCGCGGA
ShotABDF2	GGCCGCCCGCGATGCCATTCAGAAGAAGAC
ShotABDR2	AACGTTCGAGGTCGACTCTAGAGTTACGGGAACACATCGTACAGCGA
ShotY364FF	TCATCGCTGTTCGATGTGTTC
ShotY364FR	AATGTACGTGATGAGGGAC
ShotY364DF	TTCATCGCTGGATGATGTGTTCC
ShotY364DR	ATGTACGTGATGAGGGAC

Development • Supplementary information

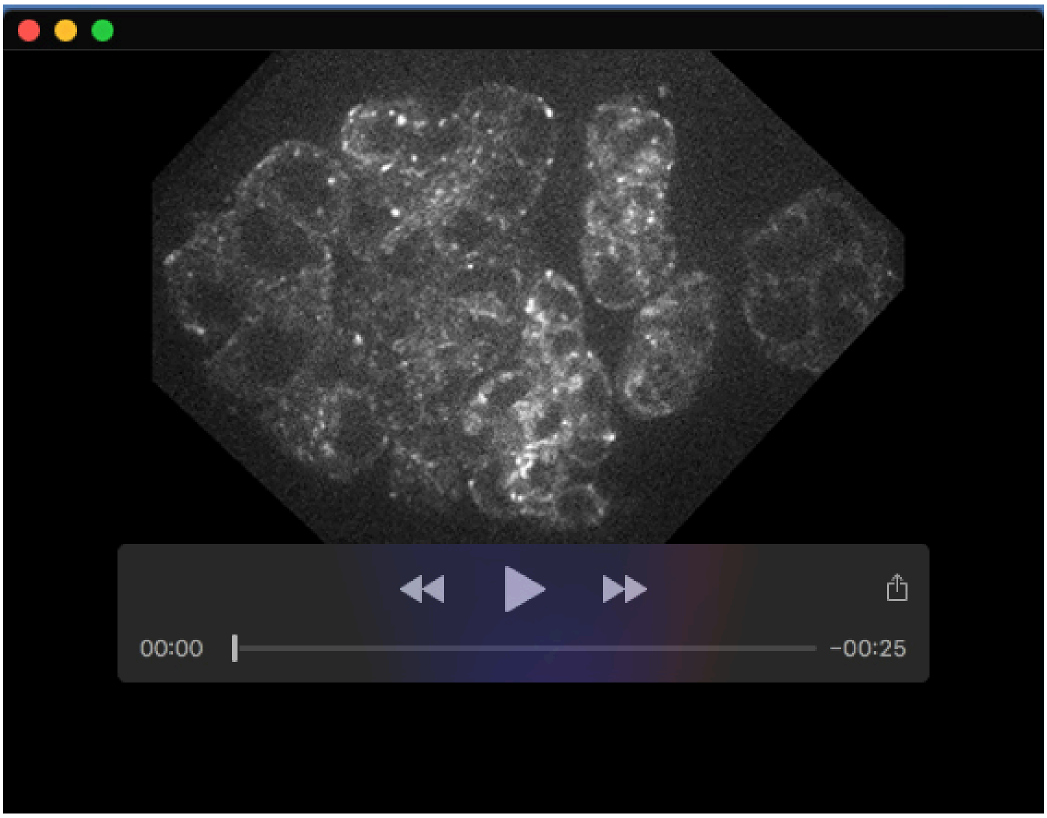

Movie 1. A time-lapse video of Shot-C-YFP in a wild-type germarium. Related to Fig. S1B. Images were collected every 1 second on a spinning disc confocal microscope. The video is shown at 15 frames/sec
