# Supplementary material for: The Shot CH1 domain recognises a distinct form of F-actin during Drosophila oocyte determination
Source: Development. 2024 Apr 2;151(7):dev202370. doi: 10.1242/dev.202370 (PMC11058685; doi:10.1242/dev.202370)
Supplement: Supplementary file 1 [file develop-151-202370_review_history.pdf]

Development

The Shot CH1 domain recognises a distinct form of F-actin during Drosophila oocyte determination

Dmitry Nashchekin, Iolo Squires, Andreas Prokop and Daniel St Johnston
DOI: 10.1242/dev.202370

Editor: Thomas Lecuit

Review timeline
Original submission: 21 September 2023
Editorial decision: 21 November 2023
First revision received: 5 February 2024
Accepted: 4 March 2024

Original submission

First decision letter

MS ID#: DEVELOP/2023/202370

MS TITLE: The Shot CH1 domain recognises a distinct form of F-actin during Drosophila oocyte determination

AUTHORS: Dmitry Nashchekin, Iolo Squires, Andreas Prokop, and Daniel St Johnston

I sincerely apologise for the delay in getting back to you. I have now received all the referees reports on the above manuscript, and have reached a decision. The referees' comments are appended below, or you can access them online: please go to BenchPress and click on the 'Manuscripts with Decisions' queue in the Author Area.

The overall evaluation is positive and we would like to publish a revised manuscript in Development, provided that the referees' comments can be satisfactorily addressed. Please attend to all of the reviewers' comments in your revised manuscript and detail them in your point-by-point response. If you do not agree with any of their criticisms or suggestions explain clearly why this is so. If it would be helpful, you are welcome to contact us to discuss your revision in greater detail. Please send us a point-by-point response indicating your plans for addressing the referees' comments, and we will look over this and provide further guidance.

Reviewer 1

Advance summary and potential significance to field

Nashchekin and colleagues investigate the link between the spectraplakin Shot and the fusome, a link that organizes MT networks for specification of the Drosophila oocyte. Through deletion and minimal constructs, they find that the actin binding domain of Shot is necessary and sufficient for localization to the fusome. However, both the actin binding domain and a separate MT binding region are needed for oocyte specification, implicating Shot as a bridge between fusome actin and the MT network organized by the fusome. However, the authors find that the actin network of the fusome has unique properties, as it is not detected by an array of different F-actin domains and only appears to be detected with actin antibodies after a specialized fixation procedure (a detection that is lost in hts mutants that lack the fusome). The authors further pursue how the actin binding domain is recruited to the fusome by investigating its CH1 and CH2 domains. These

© 2024. Published by The Company of Biologists under the terms of the Creative Commons Attribution License (https://creativecommons.org/licenses/by/4.0/).

results indicate that the CH1 domain mediates the fusome recruitment, which is modified by the CH2 domain, a nearby phosphorylation site as well as other domains of the protein. Overall, the paper advances the understanding of how the Drosophila oocyte is specified, and this advancement is important because this is the best understand model of oocyte specification. The data are sufficiently clear and properly controlled (one concern below) and replicated. The text is well written and the arguments are logical. However there is a major concern affecting the main conclusion of the paper.

Comments for the author

Major concern:
The CH1 domain may or may not be binding F-actin on the fusome for its recruitment. It remains possible that the CH1 domain could be binding a different partner for the recruitment. To test the role of F-actin in the recruitment, the effect of F-actin depolymerization on the localization of the Shot actin binding domain construct could be tested. Also, the authors refer to published structural studies of the CH1-F-actin interaction. CH1 residues mediating the F-actin interaction in the structural studies could be mutated in the Shot actin binding domain construct to test if they are required for its localization to the fusome. Experiments along these lines would be needed to clarify the role of F-actin in recruiting Shot to the fusome.

Additional concern and suggestion:
1. Only one of many approaches provides evidence of F-actin at the fusome. The antibody staining of actin, relying on an unusual fixation procedure, could be non-specific. Is this staining lost following a perturbation of F-actin depolymerization? Showing that the antibody staining of actin is dependent on F-actin is an important control for concluding that F-actin is present on the fusome.

2. A sequence comparison of the CH domains analyzed could be an informative addition. Are Shot CH1 amino acid residues implicated in mediating its F-actin interaction in the structural studies absent in the CH domains that fail to localize to the fusome?

Typos:
L85: “spektraplakins”
Line 172: “LiveAct”
Line 268: “seats”
In multiple places, “MT” should be “MTs”.
In multiple places, “a” “b” and “d” should be replaced with the symbols for alpha, beta, and delta.

Reviewer 2

Advance summary and potential significance to field

In this short manuscript, Nashchekin and colleagues performed a structure-function analysis of Spectraplakin during Drosophila oogenesis. Spectraplakin can interact with both actin and microtubule cytoskeletons, using distinct domains at the N- and C-terminus respectively. Spectraplakin or Shot in Drosophila is thus an interesting candidate that can link actin-associated fusome and microtubule-dependent oocyte determination.
Their results show that the actin-binding domains are required to bind a “specific”-type of actin on the fusome, and that the microtubule-binding domains are required for oocyte determination. Spectraplakin is a long protein with many different domains, and it is a challenge to perform such analysis in vivo. An impressive number of constructs have been generated for this study and made into transgenic flies. The images and data are convincing and interpreted rigorously. I have no major criticism regarding the experiments. Nonetheless, I suggest a few experiments that may improve the manuscript.

Comments for the author

I suggest a few experiments that may improve the manuscript.

Suggested experiments:

© 2024. Published by The Company of Biologists under the terms of the Creative Commons Attribution License (https://creativecommons.org/licenses/by/4.0/).

1) I would encourage the authors to try long incubation times with phalloidin (overnight and regular 4%PFA fixation procedure) to reveal a potential staining of this type of actin on the fusome. It would test the authors' strong conclusion on actin being different when associated with the fusome.

2) The authors proposed a model for oocyte determination in which "a slight excess of patronin.." (L70) could be sufficient to specify the oocyte. Could the overexpression of the actin- or microtubule-binding domains of Shot be sufficient to perturb oocyte determination? Would the overexpression of the actin-binding domain saturate binding sites on the fusome? Similarly, can the overexpression of C-terminal parts saturate patronin or microtubules binding sites? It would be easy to test if these domains are sufficient to delay or disrupt oocyte specification.

3) It is not always clear, which construct rescues oocyte determination.
Could the authors add that information for example in Table S1.

Suggested text changes:
1) L21 in the Abstract: "As in mammals, only one cell in a Drosophila multicellular female germline cyst is specified as an oocyte." It is actually not true, several oocytes can be specified per germline cyst in mice (Niu and Spradling, Cell, 2022).

Reviewer 3

Advance summary and potential significance to field

The manuscript entitled "The Shot CH1 domain recognises a distinct form of F-actin during Drosophila oocyte determination" by Nashchekin et al., addresses the role of different domains of the Shot protein in oocyte determination in Drosophila. In most animals, the oocyte is part of a group of cells (the cyst) from which it differentiates. How a cell gets selected to become the oocyte is unknown in most systems. This step is better understood in Drosophila than in other organisms, since it has been shown that oocyte differentiation depends on the presence of a polarized microtubule network between the cyst cells, allowing the transport of oocyte determinants to one specific cell. The formation of this network depends on the ability of the Shot protein to bind the fusome, a structure composed of endoplasmic reticulum and proteins such as Spectrins. Due to the pattern of cyst formation, the fusome is more abundant in one cell, thereby allowing the asymmetric localisation of Shot between different cyst cells. This manuscript presents new data about the identification of the different Shot domains implicated in fusome binding and in oocyte determination.

Shot contains an actin-binding domain with two calponin homology domains (CH1 and CH2) at the N-terminal region and a microtubule binding module, also composed of two domains, at the C-terminal region. The actin-binding domain and the microtubule binding module are connected by a rod domain. The authors generated different structure-function constructs that they overexpressed in a WT or in a shot background using a nanos-Gal4 driver. They first show that the N-terminal part of Shot, containing the acting binding domain, is necessary and sufficient to bind the fusome. Previously, the presence of fusomal actin was uncertain, because the main protocol for detecting Actin never gave convincing results. In this manuscript, the authors have developed a novel protocol to monitor Actin. Their results suggest that Actin is present, but in a different form, and that the spatial organisation between the Shot CH1 and CH2 domains is important for binding. Additional results show that some parts of the rod domain are also required for binding. Interestingly, the new form of actin is not recognised by most actin-binding proteins and all the classical Actin markers. Finally, the authors also demonstrate that both the ABD and the MT-binding modules are important for oocyte specification, indicating an interplay between the Shot actin-binding and MT-binding domains in organizing the microtubule network.

The manuscript is well-written (with the notable exception of figure legends – see below!) and all conclusions are supported by the data. The authors tested a large variety of molecular combinations to pinpoint the putative roles of each in fusome recognition and oocyte determination and have succeeded in clearly establishing the importance of the CH1 domain and the presence of a peculiar form of F-actin.

© 2024. Published by The Company of Biologists under the terms of the Creative Commons Attribution License (https://creativecommons.org/licenses/by/4.0/).

Overall, this manuscript is of broad interest, but in order to make it accessible to non-specialists in Drosophila oogenesis, I would strongly recommend that the authors improve the quality of figure legends and that they label the panels (arrows etc.) for clarity. This is my single key concern.

Comments for the author

Legends:
- The authors should clearly state what the object of study is in each panel (Germaria, Schematic representation, markers, constructs etc…) instead of telling the reader what they should conclude from these observations (which is in the text).
- The authors should state that the enlargement on the right is a magnified view of a fusome (and not the fusome, as there are several of them), and indicate the link with the little box in the large panel.
- The miniscule arrow-like objects (e.g. in Fig. 2) are difficult to see. Additionally, they are neither arrows nor arrowheads, and wind up looking like white specs next to the germaria. Maybe go with one or the other and increase their size?
- Line 698: Incomplete sentence: "Utrophin ABD- GFP (B), Filamin-GFP (C) and Fimbrin-YFP (D) localise to ring canals and the cell cortex but not recognise the fusome."
- In the top part of figure 4 the representation of the different regions of the germarium are incorrectly positioned relative to even the first row of germaria, let alone those lower down in the figure, rendering them entirely useless. I suggest the authors find a way to increase accuracy (positioning) and visibility (size) or delete it.

Minor comments:
For clarity, I would suggest presenting the implications of the Tyr364 mutations in the full-length constructs alongside the results of these mutations in the ABD constructs (i.e. adding lines 286 – 291 to the paragraph ending on line 259). Similarly, the results for CH1 and CH2 in full-length Shot could be added after the paragraph ending line 284.

A few typos:
- Line 294: "ShotDCH2" should be "ShotΔCH2"
- Line 332: "full-length ShotDABD" should be "full-length ShotΔABD"
- Line 670: "…that directs dynein transport of oocyte fate determinates into one cell that becomes the oocyte." Should that read determinants?

First revision

Author response to reviewers' comments

Response to reviewer's comments:

Reviewer 1 *Comments for the Author:*
Major concern:
The CH1 domain may or may not be binding F-actin on the fusome for its recruitment. It remains possible that the CH1 domain could be binding a different partner for the recruitment. To test the role of F-actin in the recruitment, the effect of F-actin depolymerization on the localization of the Shot actin binding domain construct could be tested. Also, the authors refer to published structural studies of the CH1-F-actin interaction. CH1 residues mediating the F-actin interaction in the structural studies could be mutated in the Shot actin binding domain construct to test if they are required for its localization to the fusome. Experiments along these lines would be needed to clarify the role of F-actin in recruiting Shot to the fusome.

Following the reviewer's suggestion to clarify the role of F-actin in recruiting Shot to the fusome, we used the hypomorphic *shot* allele, $shot^{2A2}$. This allele contains a point mutation in the CH1 domain that changes a conserved Val224 to Asp. According to structural studies on CH1 domains, Val224 mediates a direct interaction with F-actin. In agreement with this, the $shot^{2A2}$ mutant

© 2024. Published by The Company of Biologists under the terms of the Creative Commons Attribution License (https://creativecommons.org/licenses/by/4.0/).

reduces Shot's binding to the actin cortex in epithelia cells and stage 9 oocytes (Nashchekin et al., 2016). We now show that *shot*^{2A2} almost completely eliminates the localisation of Shot to the fusome, demonstrating that the Shot- actin interaction is required for its fusomal localisation. These data are presented in Figure 1J.

Additional concern and suggestion:
1. Only one of many approaches provides evidence of F-actin at the fusome. The antibody staining of actin, relying on an unusual fixation procedure, could be non-specific. Is this staining lost following a perturbation of F-actin depolymerization? Showing that the antibody staining of actin is dependent on F- actin is an important control for concluding that F-actin is present on the fusome.

Following reviewer 2's suggestion, we tried long incubations of ovaries with Phalloidin. This revealed that fusomal F-actin can be detected by Phalloidin after prolonged staining. To remove any uncertainties about specificity of the anti-actin antibody raised by the reviewer, we decided to substitute the anti- actin antibody data presented in Figure 3A with the long Phalloidin staining. We hope that this substitution strengthens our conclusion that F-actin is present on the fusome.

2. A sequence comparison of the CH domains analyzed could be an informative addition. Are Shot CH1 amino acid residues implicated in mediating its F-actin interaction in the structural studies absent in the CH domains that fail to localize to the fusome?

We agree with the reviewer that finding how the CH1 domains of various proteins bind different F-actin structures is an intriguing question and we are working in this direction. We have performed the suggested comparison of CH1 domains (see the alignment below). There are indeed several residues (marked by asterisks) that could potentially be involved in discriminating between F-actin networks. However, since most of these are located outside of the predicted actin binding surfaces (ABS), more comprehensive structural modelling would be required to draw any conclusions. At this stage we believe that the alignment by itself does not add much to the paper.

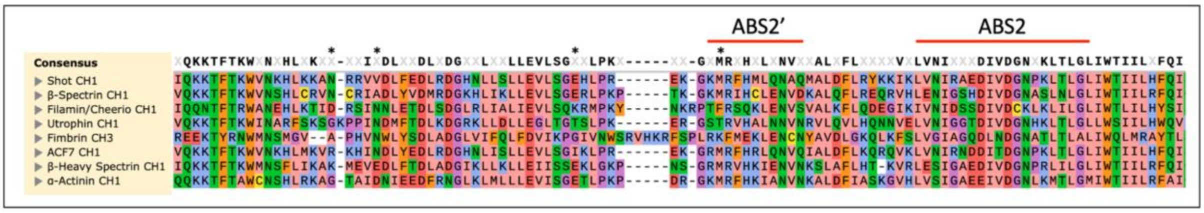

Typos:
L85: "spektraplakins"
Line 172: "LiveAct"
We have corrected this.

Line 268: "seats"
We could not find the typo.

In multiple places, "MT" should be "MTs".
In multiple places, "a" "b" and "d" should be replaced with the symbols for alpha, beta, and delta.
Changed

***Reviewer 2** Comments for the Author:*
I suggest a few experiments that may improve the manuscript.

Suggested experiments:
1) I would encourage the authors to try long incubation times with phalloidin (overnight and regular 4%PFA fixation procedure) to reveal a potential staining of this type of actin on the fusome. It would test the authors' strong conclusion on actin being different when associated with

© 2024. Published by The Company of Biologists under the terms of the Creative Commons Attribution License (https://creativecommons.org/licenses/by/4.0/).

the fusome.

Following the reviewer suggestion, we stained ovaries with Phalloidin for 21 hours after regular 4% PFA fixation. As shown at Figure 3A, it revealed that indeed after such long incubation a very weak Phalloidin signal could be detected on the fusome. It confirms the presence of F-actin on the fusome. However, considering that Phalloidin signal on the fusome is much weaker than on other actin structures in the cyst, it does not undermine our original suggestion that actin on the fusome is somehow structurally different and that prevents Phalloidin to bind it with a high efficiency. Since Phalloidin is a more reliable marker for F-actin than anti-Actin antibody, and following concerns by the reviewer 1, we substituted anti-Actin antibody staining at Figure3A with prolonged Phalloidin staining.

2) The authors proposed a model for oocyte determination in which "a slight excess of patronin.." (L70) could be sufficient to specify the oocyte. Could the overexpression of the actin- or microtubule-binding domains of Shot be sufficient to perturb oocyte determination? Would the overexpression of the actin-binding domain saturate binding sites on the fusome? Similarly, can the overexpression of C-terminal parts saturate patronin or microtubules binding sites? It would be easy to test if these domains are sufficient to delay or disrupt oocyte specification.

Following the reviewer's suggestion, we overexpressed UASP Shot ABD and UASP Shot EF-Gas2 in germline cysts using nanos-Gal4 at 29C. We could not *detect any delay or disruption in oocyte specification judging by the distribution of C(3)G (a meiotic marker that accumulates in the future oocyte):*

Accumulation of C(3)G in the cysts

Genotype	Region 3		Region 2b		
	In 1 cell	In 2 cells	In 1 cell	In 2 cells	In 3 cells
Wild type	100% (n=11)	0	41% (n=7)	47% (n=8)	12% (n=2)
Shot ABD	100% (n=10)	0	47% (n=8)	47% (n=8)	6% (n=1)
Shot EF-Gas2	100% (n=10)	0	50% (n=9)	44% (n=8)	6% (n=1)

Considering that Shot localisation in the cysts was also not affected, we suspect that overexpression of the Shot ABD does not saturate all Shot actin binding sites on the fusome. This is perhaps not surprising, as an F-actin filament can contain hundreds of actin dimers, to which the ABD can bind.
Similarly, the EF-Gas2 domain mainly binds to the lattice of MTs, which provide many potential binding sites, whereas Patronin binds to MT minus ends.

3) It is not always clear, which construct rescues oocyte determination. Could the authors add that information for example in Table S1.

We have added this information in Table S1

Suggested text changes:
1) L21 in the Abstract: "As in mammals, only one cell in a Drosophila multicellular female germline cyst is specified as an oocyte." It is actually not true, several oocytes can be specified per germline cyst in mice (Niu and Spradling, Cell, 2022).

We have change the sentence as following: "In *Drosophila* only one cell in a multicellular female germline cyst is specified as an oocyte and a similar process occurs in mammals."

Reviewer 3 *Comments for the Author:*
Legends:

© 2024. Published by The Company of Biologists under the terms of the Creative Commons Attribution License (https://creativecommons.org/licenses/by/4.0/).

- *The authors should clearly state what the object of study is in each panel (Germaria,* Schematic representation, markers, constructs etc…) instead of telling the reader what they should conclude from these observations (which is in the text).

Modified

- *The authors should state that the enlargement on the right is a magnified view of a fusome (and not the fusome, as there are several of them),*

Corrected

and indicate the link with the little box in the large panel.

We prefer to not add a link because we think that this would obscure the images. Instead, a small box in the large panel indicates the region that is enlarged on the right. We have added the following texts to the legend for Figure 3 and Figure 4 to make this clear: " The righthand panels show enlargements of the boxed regions of the fusome in the lefthand panels."

- *The miniscule arrow-like objects (e.g. in Fig. 2) are difficult to see. Additionally, they are neither arrows nor arrowheads, and wind up looking like white specs next to the germaria. Maybe go with one or the other and increase their size?*

These have been enlarged

- *Line 698: Incomplete sentence: "Utrophin ABD- GFP (B), Filamin-GFP (C) and Fimbrin-YFP (D) localise to ring canals and the cell cortex but not recognise the fusome."*

We have corrected this sentence

- *In the top part of figure 4 the representation of the different regions of the germarium are incorrectly positioned relative to even the first row of germaria, let alone those lower down in the figure, rendering them entirely useless. I suggest the authors find a way to increase accuracy (positioning) and visibility (size) or delete it.*

We have deleted the diagram in the top part of Figure 4

Minor comments:
For clarity, I would suggest presenting the implications of the Tyr364 mutations in the full- length constructs alongside the results of these mutations in the ABD constructs (i.e. adding lines 286 – 291 to the paragraph ending on line 259). Similarly, the results for CH1 and CH2 in full-length Shot could be added after the paragraph ending line 284.

We have rearranged the text as requested.

A few typos:
- *Line 294: "ShotDCH2" should be "ShotΔCH2"*
- *Line 332: "full-length ShotDABD" should be "full-length ShotΔABD"*
- *Line 670: "…that directs dynein transport of oocyte fate determinates into one cell that becomes the oocyte." Should that read determinants?*

These have been corrected

<u>Second decision letter</u>

MS ID#: DEVELOP/2023/202370

© 2024. Published by The Company of Biologists under the terms of the Creative Commons Attribution License (https://creativecommons.org/licenses/by/4.0/).

MS TITLE: The Shot CH1 domain recognises a distinct form of F-actin during Drosophila oocyte determination

AUTHORS: Dmitry Nashchekin, Iolo Squires, Andreas Prokop, and Daniel St Johnston
ARTICLE TYPE: Research Article

I am happy to tell you that your manuscript has been accepted for publication in Development, pending our standard ethics checks.

Reviewer 1

Advance summary and potential significance to field

The authors have effectively addressed my past concerns. This advance to the understanding of Drosophila oocyte determination should be of interest to the readers of Development.

Comments for the author

No further suggestions.

Reviewer 2

Advance summary and potential significance to field

The authors have added only one panel with the suggested phalloidin protocol.
Otherwise, they have answered to all my comments.

Comments for the author

The authors have answered to all my comments

Reviewer 3

Advance summary and potential significance to field

The manuscript entitled “The Shot CH1 domain recognises a distinct form of F-actin during Drosophila oocyte determination” by Nashchekin et al., addresses the role of different domains of the Shot protein in oocyte determination in Drosophila. In most animals, the oocyte is part of a group of cells (the cyst) from which it differentiates. How a cell gets selected to become the oocyte is unknown in most systems. This step is better understood in Drosophila than in other organisms, since it has been shown that oocyte differentiation depends on the presence of a polarized microtubule network between the cyst cells, allowing the transport of oocyte determinants to one specific cell. The formation of this network depends on the ability of the Shot protein to bind the fusome, a structure composed of endoplasmic reticulum and proteins such as Spectrins. Due to the pattern of cyst formation, the fusome is more abundant in one cell, thereby allowing the asymmetric localisation of Shot between different cyst cells. This manuscript presents new data about the identification of the different Shot domains implicated in fusome binding and in oocyte determination.

Comments for the author

Based on the new data presented in the manuscript and on the responses to our suggestions, I fully favor publication of this work in Development. I consider that the authors provide satisfactory explanations to the reviewer’s comments. I also appreciated the new organization of some of the data and of the figures. The publication of this manuscript will surely be of importance for people in the field.

© 2024. Published by The Company of Biologists under the terms of the Creative Commons Attribution License (https://creativecommons.org/licenses/by/4.0/).
